# OA08.04. Brain circuitry subserving acupuncture relief of itch in atopic dermatitis: an fMRI study

**DOI:** 10.1186/1472-6882-12-S1-O32

**Published:** 2012-06-12

**Authors:** V Napadow, A Li, M Loggia, J Kim, P Schalock, E Lerner, B Rosen, T Kaptchuk, F Pfab

**Affiliations:** 1 Harvard Medical School, Massachusetts General Hospital, Martinos Center, Charlestown, USA; 2 Department of Dermatology, Massachusetts General Hospital, Boston, USA; 3 Department of Medicine, Beth Israel Hospital, Boston, USA

## Purpose

Chronic itch is a prevalent symptom of many inflammatory skin disorders, including atopic dermatitis (AD). While conventional systemic approaches to reduce AD itch have shown limited efficacy and/or significant side effects, several recent studies have demonstrated effectiveness of acupuncture for reducing itch in healthy adults and AD. We sought to evaluate the brain circuitry underlying acupuncture reduction of itch.

## Methods

We evaluated n=14 AD patients (age: 25.4±9.1yrs) showing type-I-sensitivity to grass pollen, Dermatophagoides farinae or D. pteronyssinus. A previously validated itch modulation model was used to create a block design paradigm in conjunction with fMRI (3T, Siemens Trio, Germany). Itch was induced with subject-specific allergen prick testing, and its intensity experimentally increased and decreased using a thermode (Medoc, Israel). Brain response to itch was investigated before and after (1) real (100Hz EA at LI-11 to HT-3) and (2) sham acupuncture, as well as (3) antihistamine (levocetirizine) and (4) placebo solution. This was a crossover study where each patient experienced each therapy in separate randomized MRI scan sessions.

## Results

Clinically-relevant allergen itch produced activation in anterior insula, putamen, and ventrolateral prefrontal gyri. Real, but not sham, acupuncture, and neither anti-histamine nor placebo solution were found to reduce itch sensation (ACUP: base=66±18, post=44±18, p<0.001). Following real acupuncture, there was diminished itch-evoked brain activity in right aIns, putamen, and nucleus accumbens. Furthermore, insula, putamen, and S2 response to acupuncture correlated with reduced itch ratings; thereby linking brain response to acupuncture stimuli with clinical outcomes.

## Conclusion

Putamen response may reflect affective/motivational aspects of itch, consistent with the urge to scratch. Down-regulation of salience (insula) and affective/motivational (putamen) components of itch in AD may underlie acupuncture efficacy in this clinical population. Based on our data, acupuncture protocols which activate insula and S2, but not putamen should optimize itch reduction. This neuroimaging biomarker should be further explored.

